# Qici Sanling Decoction Suppresses Glutamine Consumption and Bladder Cancer Cell Growth through Inhibiting c-Myc Expression

**DOI:** 10.1155/2022/7985468

**Published:** 2022-01-11

**Authors:** Weihua Chen, Weifeng Wang, Jun Zhang, Guoqiang Liao, Jie Bai, Bo Yang, Mingyue Tan, Hua Gong

**Affiliations:** ^1^Department of Urology, Shanghai East Hospital, Tongji University, Shanghai 200120, China; ^2^Department of Urology, Shanghai University of Medicine & Health Sciences Affiliated Zhoupu Hospital, Shanghai 201318, China; ^3^Graduate School, Shanghai University of Traditional Chinese Medicine, Shanghai 201318, China; ^4^Department of Urology, Shuguang Hospital, Shanghai University of Traditional Chinese Medicine, Shanghai 201203, China; ^5^Department of Urology, Shanghai General Hospital, Shanghai Jiao Tong University School of Medicine, Shanghai 200080, China

## Abstract

Traditional Chinese medicine (TCM) is widely used as an alternative therapy for cancer treatment in China. Glutamine catabolism plays an important role in cancer development. Qici Sanling decoction (QCSL) suppresses bladder cancer growth. However, the association between QCSL and glutamine catabolism remains unknown. In this study, different doses of QCSL were applied to T24 cells, followed by the measurements of cell viability and apoptosis using CCK-8 and Annexin V/PI assay, respectively. Furthermore, glutamine consumption was detected using the glutamine assay kit. QCSL was observed to inhibit cell growth and induced cell apoptosis in a dose-dependent manner. Analysis of glutamine consumption revealed that QCSL suppressed glutamine consumption in T24 cells. Furthermore, QCSL decreased the mRNA and protein levels of c-Myc, GLS1, and SLC1A5. All these effects induced by QCSL could be alleviated by c-Myc overexpression, indicating c-Myc was involved in the protective role of QCSL in bladder cancer. In addition, QCSL was found to inhibit tumor growth in the xenograft tumor model. The similar results were obtained in tumor samples that protein levels of c-Myc, GLS1, and SLC1A5 were decreased upon treatment with QCSL. In conclusion, QCSL suppresses glutamine consumption and bladder cancer cell growth through inhibiting c-Myc expression.

## 1. Introduction

Bladder cancer (BC) is one of the most common malignancies, which accounts for an estimated 500,000 new cases and 200,000 deaths each year throughout the world [1]. The high recurrence rate is the main cause of death. Cigarette smoking is considered as the main risk of factor, but advanced age, *Schistosoma haematobium* infection, and male sex also contribute to the development of BC [2]. Recently, the tumor microenvironment (TME) attracts researchers' attentions as playing a key role in tumor development, including BC. The diverse TME observed in tumor indicates that adequate nutrients are needed for growth of tumor cells. Glutamine, one of the most abundant amino acids in the TME, is of great importance in the cancer cell metabolism and proliferation [3].

To fulfill the rapid proliferation of cancer cells, enhancing the carbon flux through glutaminolysis is observed [4]. Glutaminolysis is the process of glutamine catabolism. First, glutamine is catalyzed by glutaminase (GLS) to generate glutamate. Then, glutamate dehydrogenase (GDH) converts glutamate to *α*-ketoglutarate (*α*-KG), which is the intermediate of tricarboxylic acid (TCA) cycle [5]. Glutaminase 1 (GLS1) and glutaminase 2 (GLS2) are the two types of GLS. GLS1, frequently upregulated in tumors, is regulated by the transcription factor c-Myc [6]. c-Myc is a member of the Myc family which plays critical roles in numerous cellular processes [7]. c-Myc has been reported to activate the expression of glutaminase, GLS, and SLC1A5 (solute carrier family 1 member 5), resulting in the regulation of glutamine uptake and catabolism [8].

Qici Sanling decoction (QCSL), a widely used traditional Chinese medicine (TCM) in China, is made of *Astragalus propinquus* Schischkin (Fabaceae) (Huangqi), *Sagittaria sagittifolia* L. (Alismataceae) (Cigu), *Polyporus umbellatus* Huds (Polyporus) (Zhuling), *Poria cocos* Wolf (Polyporaceae) (Fuling), *Radix paeoniae* Alba (Ranunculaceae) (Baishao), *Curcuma longa* L. (Zingiberaceae) (Ezhu), *Cinnamomi ramulus* Blume (Lauraceae) (Guizhi), *Glycyrrhiza glabra* L. (Febaceae) (Gancao), *Rehmannia glutinosa* L. (Phrymaceae) (Shudi), and *Glabrous greenbrier* rhizome (Liliaceae) (Tufuling) at a ratio of 10 : 10 : 5 : 5 : 5 : 5 : 3 : 2 : 5 : 5 in dry weight. In our previous study [9], we reported that Qici Sanling decoction suppressed bladder cancer growth by inhibiting the Wnt/*β*-catenin pathway. In this study, we focused on the glutamine metabolism involved in the process that QCSL inhibits cell growth in bladder cancer.

## 2. Materials and Methods

### 2.1. Cell Culture

Human bladder cancer cell line T24 was obtained from Cell Bank of the Chinese Academy of Sciences (Shanghai, China) and cultured in the RPMI-1640 medium, supplemented with 10% FBS, penicillin (100 U/mL), and streptomycin (100 *μ*g/mL), in a 5% CO_2_ humidified incubator at 37°C.

### 2.2. Preparation of QCSL and Lentivirus

QCSL was purchased from E-Fong Pharmaceutical (Guangdong, China) and dissolved with PBS as previously described [9]. Overexpressing lentivirus targeting c-Myc was purchased from GeneChem Company (Shanghai, China).

### 2.3. CCK-8 Cell Viability Assay

T24 cells were planted 3000 per well into a 96-well plate and cultured overnight in an incubator. Different doses (0, 0.05, 0.1, 0.2, 0.5, 1, and 2 mg/ml) of QCSL were applied to T24 cells. After incubation for 12, 24, and 48 h, 10 *μ*l of CCK-8 (Beyotime, Shanghai, China) solution was added into each well and cultured for 1 h. Absorbance at 450 nm was measured using a microplate reader (BioRad Laboratories, Inc., Hercules, CA, USA).

### 2.4. Cell Apoptosis Assay

T24 cells were planted into a 6-well plate and cultured overnight in an incubator; then, QCSL were applied to T24 cells. After 48 h incubation, all the cells including the culture medium were collected according to the instruction of the Annexin V/PI cell apoptosis detection kit (BioRad Laboratories). Cells were stained with Annexin V for 5 min in the dark, followed by staining with PI for 2 min. Finally, cell apoptosis was analyzed using flow cytometry.

### 2.5. Glutamine Consumption

Glutamine concentration of each group was measured using the glutamine assay kit (Abcam) following the manufacturers' instructions. Glutamine consumption was the glutamine concentration in the original medium minus that in the treated medium.

### 2.6. Western Blot

Total protein from treated cells and tumor samples from the xenograft tumor model was extracted using RIPA buffer (BioRad Laboratories) and separated according to standard procedures of SDS-PAGE. Primary antibody including anti-c-Myc (1 : 1000, ab32072, Abcam, USA), anti-GLS1 (1 : 1000, ab156876, Abcam), anti-SLC1A5 (1 : 1000, ab237704, Abcam), anti- GAPDH (1 : 2000, #5174, CST, USA), and horseradish peroxidase (HRP)-conjugated secondary antibodies (Beyotime) were used to incubate membranes. Finally, the proteins were visualized using an ECL solution (Millipore, USA) and imaged using a Tanon-5200 Multisystem (Tanon, Shanghai, China).

### 2.7. Real-Time PCR

Total RNA of treated cells was extracted using TRIzol reagent (Invitrogen, Carlsbad, CA). The reverse transcription system (TaKaRa, Dalian, China) and SYBR Green qPCR Mixes (TaKaRa) were used to measure gene expression. The reactions were performed on a 7900HT Sequence Detection System (Applied Biosystems, USA). Relative gene expression level was analyzed using the comparative Ct method (2^−ΔCt^). The primers used are as follows:

  c-Myc: primer F 5′ ATCCTGTCCGTCCAAGCA 3′, primer R 5′ CGCACAAGAGTTCCGTAG 3′
  GLS1: primer F 5′ CTGTGCTCCATTGAAGTG 3′, primer R 5′ TGCCCTGAGAAGTCATAC 3′
  SLC1A5: primer F 5′ ATCCATGGGCTCCTGGTACT 3′, primer R 5′ CACGCACTTCATCATCAGCG 3′
  GAPDH: primer F 5′ AATCCCATCACCATCTTC 3′, primer R 5′ AGGCTGTTGTCATACTTC 3′

### 2.8. Xenograft Tumor Model

BALB/c-nu nude mice (4–6 weeks) were purchased from Shanghai Laboratory Animal Research Center (Shanghai, China). All mice were housed at 20–23°C in a 12 h light/dark cycle with food and tap water supplied ad libitum. Xenograft tumor models were established as previously described [9]. After 2 weeks, 18 mice were randomly divided into three groups (vehicle, 200 mg/kg QCSL, and 400 mg/kg QCSL). QCSL was applied to the mice in QCSL groups by gavage once a day for 3 weeks, and the mice in the vehicle group was given the same amount of PBS. Then, the mice were anesthetized by isoflurane. The tumor samples from each group were fixed in 10% (v/v) formalin for TUNEL examination and frozen for Western blot. The experimental procedures for nude mouse xenograft tumor models and daily care were approved by the Committee on Ethical Use of Animals of Shanghai University of Traditional Chinese Medicine. All experiments were performed in accordance with the national legislation and with the National Institutes of Health Guidelines regarding the care and use of animals for experimental procedures.

### 2.9. TUNEL Assay

After 48 h fixation in 10% (v/v) formalin, tumor tissues were paraffin embedded and cut into sections (5 mm; Leica RM2125, Germany). Cell apoptosis of tumor tissues were detected using a TUNEL Kit (Roche, Indianapolis, IN, USA) following the manufacturer's instructions. Percentages of apoptotic cells were evaluated in five randomly selected fields.

### 2.10. Statistical Analysis

Data were presented as the mean ± standard deviation. One-way analysis of variance (ANOVA) was used to compare the differences between 3 or more groups. Differences between two groups were determined by Student's *t*-test. In addition, *P* < 0.05 was considered statistically significant.

## 3. Results

### 3.1. QCSL Inhibits Cell Viability and Induces Cell Apoptosis in BC Cells

**Figure 1:**
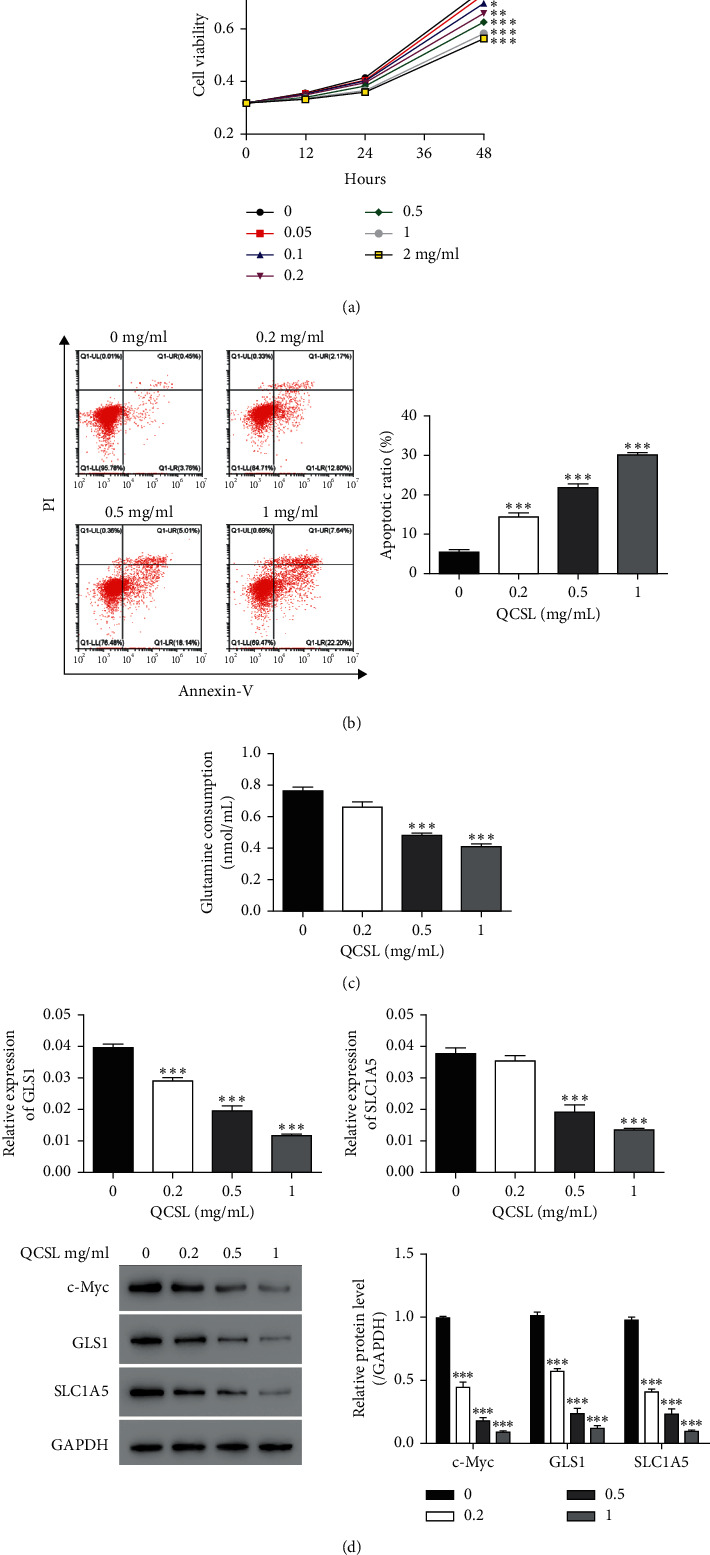
QCSL inhibits cell viability and glutamine catabolism. (a) The viability of T24 cells treated with different concentrations (0, 0.05, 0.1, 0.2, 0.5, 1, and 2 mg/ml) of QCSL measured by CCK-8. (b) Cell apoptosis assay performed after treatment with different concentrations (0, 0.2, 0.5, and 1 mg/ml) of QCSL. (c) Glutamine consumption was detected after the same treatment as in b. (d) The mRNA and protein levels of GLS1, SLC1A5, and c-Myc. ^*∗*^*P* < 0.05, ^*∗∗*^*P* < 0.01, ^*∗∗∗*^*P* < 0.001 vs. 0 mg/ml.

To explore the function of QCSL in BC, different doses (0, 0.05, 0.1, 0.2, 0.5, 1, and 2 mg/ml) of QCSL were applied to BC cell line, T24 cells. As shown in Figure 1(a), QCSL significantly inhibited cell viability in a dose-dependent manner in T24 cells and 0, 0.2, 0.5, and 1 mg/ml of QCSL were selected for the following experiments. The results indicated that all three concentrations of QCSL markedly increased the cell apoptosis (Figure 1(b)).

### 3.2. QCSL Inhibits GLS1 Expression and Glutamine Consumption

Given the critical role of glutamine catabolism in cancer cell proliferation, it is rationale to investigate whether and how QCSL regulates glutamine catabolism in BC cell line. To this end, first, glutamine consumption was measured after treatment with QCSL. As shown in Figure 1(c), glutamine consumption was significantly suppressed by QCSL. Next, we hypothesize the decreased glutamine consumption due to reduction of the GLS1 level which is a rate-limiting enzyme of glutamine catabolism. To test, we measured GLS1 mRNA and protein levels and found that GLS1 was downregulated after treatment with QCSL in T24 cells (Figure 1(d)). In addition, the mRNA and protein levels of SLC1A5 were reduced as well implicating decrease of glutamine uptake. To further investigate the mechanism contributing to reductions of GLS1 and SLC1A5, the level of c-Myc was measured and decrease of c-Myc was observed upon QCSL treatment (Figure 1(d)).

### 3.3. QCSL Increases Cell Apoptosis in T24 Cells by Repressing c-Myc Expression

**Figure 2:**
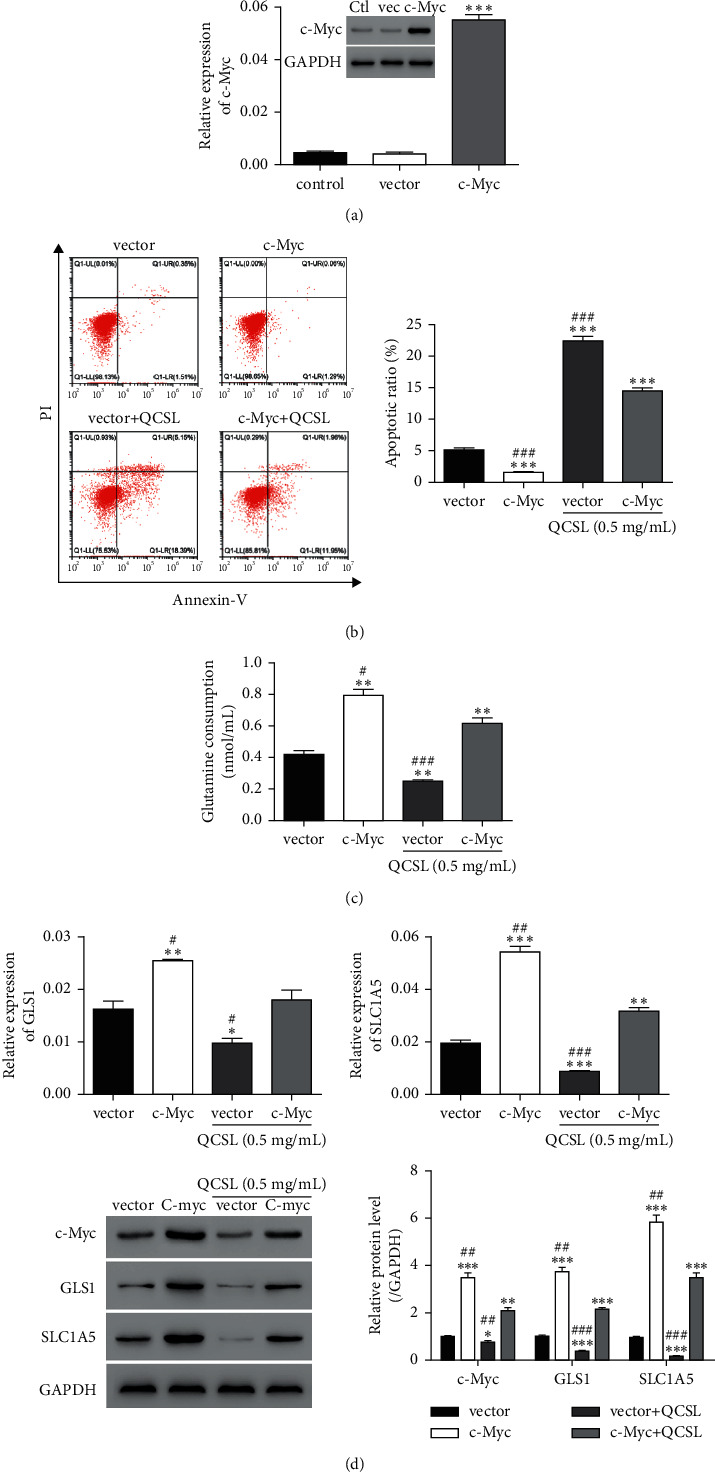
QCSL increases cell apoptosis in T24 cells by repressing c-Myc expression. (a) mRNA and protein levels of c-Myc measured after transduction with vector and c-Myc overexpressing lentivirus. ^*∗∗∗*^*P* < 0.001 vs. vector. (b) Cell apoptosis assay performed after treatment with 0.5 mg/ml of QCSL and/or c-Myc overexpressing lentivirus. (c) Glutamine consumption detected after the same treatment as in b. (d) The mRNA and protein levels of GLS1, SLC1A5, and c-Myc. ^*∗∗*^*P* < 0.01, ^*∗∗∗*^*P* < 0.001 vs. vector; ^##^*P* < 0.01, ^###^*P* < 0.001 vs. c-Myc + QCSL.

To verify our hypothesis that c-Myc involves in the function of QCSL in BC, we overexpressed c-Myc by transfecting T24 cells with c-Myc overexpressing lentivirus. As shown in Figure 2(a), mRNA and protein levels of c-Myc were significantly upregulated in the cells transduced with c-Myc overexpressing lentivirus. Based on the data shown in Figure 1, 0.5 mg/ml of QCSL was chosen for further experiments due to the obvious difference of the cell apoptotic rate but relative normal cell morphology upon the dosage treatment. The results showed that QCSL treatment could increase the cell apoptosis which can be alleviated by c-Myc overexpression (Figure 2(b)). Similarly, overexpression of c-Myc robustly increase the glutamine consumption, which can be rescued by QCSL treatment. Cotreatment with QCSL and c-Myc overexpressing lentivirus exhibited higher glutamine consumption than treatment with QCSL alone but still lower than c-Myc overexpression (Figure 2(c)). Mechanistically, the decrease of GLS1, SLC1A5, induced by QCSL treatment could be rescued by c-Myc overexpressing (Figure 2(d)). Taken together, QCSL increased cell apoptosis in T24 cells by repressing c-Myc expression.

### 3.4. QCSL Suppresses Tumor Growth in the BC Xenograft Mouse Model

**Figure 3:**
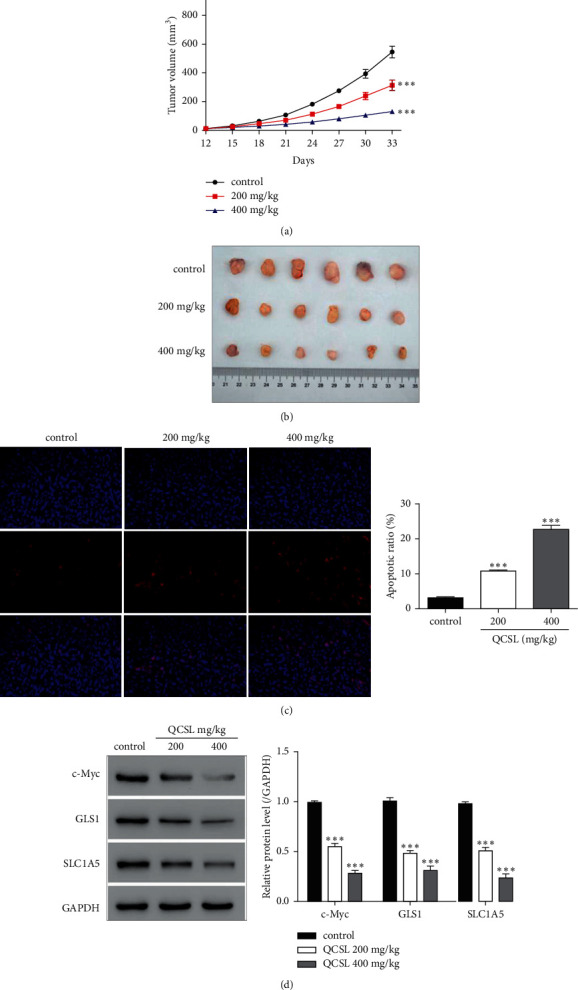
QCSL suppresses tumor growth in the BC xenograft mouse model. (a) Tumor volumes of vehicle, 200 mg/kg, and 400 mg/kg QCSL groups. ^*∗∗∗*^*P* < 0.001 vs. control. (b) Image of tumors of each group. (c) TUNEL assay performed using the tumor samples of each group. (d) The protein levels of GLS1, SLC1A5, and c-Myc measured using the tumor samples of each group. ^*∗∗∗*^*P* < 0.001 vs. vehicle.

The previous results in Figures 1 and 2 render the rationale to treat BC with QCSL in vivo. To study the tumor suppressing role of QCSL, T24 cells were injected into nude mice to construct the xenograft tumor model. As shown in Figures 3(a) and 3(b), treatment with 200 and 400 mg/kg QCSL markedly inhibited tumor growth. TUNEL results of tumor samples showed that the apoptotic cells were much more in QCSL groups than that in the control group (Figure 3(c)), indicating that QCSL induced cell apoptosis in vivo. Furthermore, the decrease of GLS1, SLC1A5, and c-Myc induced by QCSL was also found in tumor samples (Figure 3(d)). These results suggest that QCSL suppresses BC tumor growth by regulating glutamine catabolism.

## 4. Discussion

TCM has been wildly used in Asia, particularly in China for thousand years. In the present time, many TCMs were accepted to be an alternative therapy for cancer treatment. However, the major limitation for applications of TCM is the unclear mechanisms due to the complicated components of TCM. Even some of TCMs has been reported to treat bladder cancer [10–12], and the role of QCSL in therapy of bladder cancer is still unclear.

Instead of the consumption of glucose, cancer cells have also been shown to favor glutamine as a preferential fuel. The glutaminolysis metabolism plays a vital role in the tumorigenesis and the occurrence of chemoresistance. In our previous study, we reported that QCSL inhibited T24 cells growth by inactivating the Wnt/*β*-catenin pathway. Of note, QCSL treatment dramatically reduced the level of c-Myc [9]. These results render the rationale to further study the role of c-Myc in BC.

c-Myc has been reported to active the expression of glutaminase, GLS, and SLC1A5, resulting in the regulation of glutamine uptake and metabolism [8]. To study, we overexpressed c-Myc in T24 cells and observed increase of glutamine consumption upon c-Myc overexpressing (Figure 2). In addition to glutamine consumption, elevation of SLC1A5 indicates increase of glutamine uptake which makes sense that tumor cell growth requires more glutamine as well. Of note, QSCL could dramatically inhibit the level of c-Myc resulting in reduction of GLS1 and SLC1A5 due to the transcriptional effects. Reductions of GLS1 and SLC1A5 could induce decrease of glutaminolysis and glutamine uptake which will finally result in reduction of energy supplies and tumor suppression through inducing tumor cell apoptosis. Even our study supports the notion that QSCL induces cell apoptosis by preventing the glutamine metabolism via regulating c-Myc; however, how QSCL regulating c-Myc is still unclear. Translational and posttranslational regulations should be considered in the future study. On the other hand, due to the multiple facets of c-Myc, we also need to clarify the other roles of c-Myc in tumorigenesis except for the glutamine metabolism, including glucose and lipid metabolism, cell cycle, and tumor microenvironment.

The collective data from in vitro study render strong rationale for the translational study in the xenograft tumor model. In the study, we observed QSCL inhibited tumor growth in a dose-dependent manner. It is important to determine the appropriate dosage of QSLC to treat BC in balance with toxicity and best therapeutic effects. This finding provides us a new strategy to treat BC by controlling glutamine catabolism. The results also shed light on the new therapeutic strategy in combination of QSLC with glutamine catabolism inhibitor in the future. However, more studies are required before this application but anyway projects the dawn.

## 5. Conclusions

QCSL suppresses glutamine consumption and bladder cancer cell growth through inhibiting c-Myc expression.

## Data Availability

The data that support the findings of this study are available from the corresponding authors upon request.
